# Production, quality control, stability, and potency of cGMP-produced *Plasmodium falciparum* RH5.1 protein vaccine expressed in *Drosophila* S2 cells

**DOI:** 10.1038/s41541-018-0071-7

**Published:** 2018-08-17

**Authors:** Jing Jin, Richard D. Tarrant, Emma J. Bolam, Philip Angell-Manning, Max Soegaard, David J. Pattinson, Pawan Dulal, Sarah E. Silk, Jennifer M. Marshall, Rebecca A. Dabbs, Fay L. Nugent, Jordan R. Barrett, Kathryn A. Hjerrild, Lars Poulsen, Thomas Jørgensen, Tanja Brenner, Ioana N. Baleanu, Helena M. Parracho, Abdessamad Tahiri-Alaoui, Gary Whale, Sarah Moyle, Ruth O. Payne, Angela M. Minassian, Matthew K. Higgins, Frank J. Detmers, Alison M. Lawrie, Alexander D. Douglas, Robert Smith, Willem A. de Jongh, Eleanor Berrie, Rebecca Ashfield, Simon J. Draper

**Affiliations:** 1 The Jenner Institute, University of Oxford, Old Road Campus Research Building, Oxford, OX3 7DQ UK; 2 Clinical BioManufacturing Facility, University of Oxford, Oxford, OX3 7JT UK; 3 ExpreS²ion Biotechnologies, SCION-DTU Science Park, Agern Allé, 12970 Hørsholm, Denmark; 4 Department of Biochemistry, University of Oxford, South Parks Road, Oxford, OX1 3QU UK; 5 Thermo Fisher Scientific, J.H. Oortweg 21, 2333 CH Leiden, Netherlands

## Abstract

*Plasmodium falciparum* reticulocyte-binding protein homolog 5 (PfRH5) is a leading asexual blood-stage vaccine candidate for malaria. In preparation for clinical trials, a full-length PfRH5 protein vaccine called “RH5.1” was produced as a soluble product under cGMP using the ExpreS^2^ platform (based on a *Drosophila melanogaster* S2 stable cell line system). Following development of a high-producing monoclonal S2 cell line, a master cell bank was produced prior to the cGMP campaign. Culture supernatants were processed using C-tag affinity chromatography followed by size exclusion chromatography and virus-reduction filtration. The overall process yielded >400 mg highly pure RH5.1 protein. QC testing showed the MCB and the RH5.1 product met all specified acceptance criteria including those for sterility, purity, and identity. The RH5.1 vaccine product was stored at −80 °C and is stable for over 18 months. Characterization of the protein following formulation in the adjuvant system AS01_B_ showed that RH5.1 is stable in the timeframe needed for clinical vaccine administration, and that there was no discernible impact on the liposomal formulation of AS01_B_ following addition of RH5.1. Subsequent immunization of mice confirmed the RH5.1/AS01_B_ vaccine was immunogenic and could induce functional growth inhibitory antibodies against blood-stage *P. falciparum* in vitro. The RH5.1/AS01_B_ was judged suitable for use in humans and has since progressed to phase I/IIa clinical trial. Our data support the future use of the *Drosophila* S2 cell and C-tag platform technologies to enable cGMP-compliant biomanufacture of other novel and “difficult-to-express” recombinant protein-based vaccines.

## Malaria: Successful clinical trial preparation for blood-stage vaccine

A vaccine candidate for blood-stage malaria has overcome previous hurdles to enter clinical trials. The protein PfRH5 is an essential blood-stage infection facilitator of malarial parasite *Plasmodium falciparum*, and a promising target for vaccine strategies. Unfortunately, efforts to produce the protein in an immunogenic, clinically-viable way have been met with difficulty. Here, researchers led by Simon Draper, from the UK’s Jenner Institute, used a fruit fly expression system to produce over 400 mg of high-purity protein. Formulated with an immunity-boosting adjuvant, the vaccine elicited antibodies in mice that proved inhibitory to blood-stage *P. falciparum* during *in vitro* assays. The PfRH5 vaccine candidate and its adjuvant have been approved for a clinical trial in the UK, and the authors hope that the expression system used may be beneficial in the expression of other ‘difficult’ proteins.

## Introduction

Malaria caused by the parasite *Plasmodium falciparum* continues to exert a huge burden on global public health, with over 200 million clinical cases annually and approximately half a million deaths.^1^ Central to ongoing efforts to develop highly effective vaccines against malaria infection, disease, or transmission is the production of recombinant proteins for use as subunit vaccines.^2^ These vaccines seek to induce antibodies that interfere with critical steps during the parasite’s complex lifecycle—including sporozoite invasion of the liver, merozoite invasion of the red blood cell (RBC), sequestration of infected RBC, or sexual replication and midgut traversal by the parasite within the mosquito.^3^ Recombinant vaccine antigen may take numerous forms, ranging from simple peptide to soluble monomeric protein through to oligomeric scaffolds^4,5^ or larger virus-like particles (VLPs).^6–8^

Typical delivery of these vaccines requires formulation of the protein antigen with a defined chemical adjuvant^9,10^ in order to maximize quantitative antibody immunogenicity, while maintaining acceptable levels of reactogenicity and aiming to avoid detrimental impact of the adjuvant on the protein’s conformational integrity. Notable successful recombinant human vaccines include hepatitis B virus surface antigen (HBsAg) and human papillomavirus.

The blood-stage of malaria infection leads to the associated morbidity and mortality through a variety of pathological mechanisms.^11^ Vaccines targeting this stage thus aim to protect against death and clinical disease, while also combating transmission via reduction in blood-stage parasitemia. In this regard, merozoite proteins involved in RBC invasion have traditionally been targeted via the induction of growth inhibitory antibodies,^3^ however historical candidate antigens have suffered from substantial levels of polymorphism and redundancy, leading to non-protective or strain-specific vaccine-induced antibody responses.^12,13^ Recently, a number of more promising antigens have been identified that are relatively highly conserved and yet remain susceptible to neutralizing vaccine-induced antibodies.^2^ The most advanced of these candidates is the *P. falciparum* reticulocyte-binding protein homolog 5 (PfRH5).^14^

Vaccination of animals with PfRH5 induces antibodies that inhibit all *P. falciparum* lines and field isolates tested to date.^15–18^ PfRH5 is also essential,^19,20^ forming a non-redundant interaction with basigin (CD147) on the RBC surface during invasion.^21^ The high degree of PfRH5 sequence conservation is associated with low-level immune pressure following years of natural malaria exposure,^15,22–24^ coupled with functional constraints linked to basigin binding and host RBC tropism.^20,25–27^ Notably, *Aotus* monkeys were protected by PfRH5 vaccination against a stringent heterologous strain blood-stage *P. falciparum* challenge.^28^ In this study, anti-PfRH5 serum IgG antibody concentration and in vitro growth inhibition activity (GIA) measured using purified IgG were both associated with protective outcome in vivo.

Consequently, there has been strong impetus to progress PfRH5-based vaccines into early-phase clinical testing. The first reported clinical trial utilized a recombinant adenovirus-poxvirus vectored platform to deliver PfRH5 in healthy UK adults.^24^ The vaccines were shown to be safe, and led to the induction of PfRH5-specific antibodies, B-cell and T-cell responses, exceeding the serum antibody responses observed in African adults following years of natural malaria exposure. However, these serum antibody responses still only reached peak median levels of ~9 µg/mL PfRH5-specific IgG, suggesting substantial room for improvement in terms of quantitative vaccine immunogenicity. Indeed, previous malaria vaccine candidates delivered as recombinant antigen formulated in strong adjuvant (such as Alhydrogel + CPG 7909 or GlaxoSmithKline’s (GSK) adjuvant system AS01_B_) have achieved peak responses of ≥100 µg/mL antigen-specific serum IgG in humans.^29–31^

Immunization of animals with full-length PfRH5 protein antigen leads to the induction of neutralizing antibodies,^15–18^ in contrast to the earliest vaccination studies that used PfRH5 fragments made in *Escherichia coli*, which failed to induce functional antibodies.^19,32^ In order to progress clinically, it has therefore been critical to develop a protein expression and purification process that (i) allowed for production of full-length PfRH5 protein, and (ii) was scalable and compliant with current good manufacturing practice (cGMP). In this regard, we reported the production of soluble full-length PfRH5 protein using a cGMP-compliant platform called ExpreS^2^, based on a *Drosophila melanogaster* Schneider 2 (S2) stable cell line system.^33,34^ Full-length PfRH5 protein was expressed from stable S2 cell lines and secreted into the supernatant from where it was purified using a newly available affinity purification system that makes use of a C-terminal tag known as “C-tag,” composed of the four amino acids (aa) glutamic acid–proline–glutamic acid–alanine (E-P-E-A).^35^ This C-tag is selectively captured on a resin coupled to a camelid single-chain antibody specific for this short sequence^36^ that has now been developed into a CaptureSelect™ affinity resin by Thermo Fisher Scientific.

Here we describe the production, quality control, stability, and potency of a full-length PfRH5 soluble protein vaccine called RH5.1, which we produced to cGMP using the ExpreS^2^ and C-tag platform technologies at the University of Oxford’s Clinical BioManufacturing Facility (CBF). Given the limited prior use of *Drosophila* S2 cells and C-tag resin for cGMP vaccine production, this biomanufacturing process necessitated the development of new processes and consultation with the UK regulator—the Medicines and Healthcare products Regulatory Agency (MHRA). This work led to the successful production of >400 mg RH5.1 protein vaccine, which has subsequently progressed to phase I/IIa clinical testing in healthy adults in Oxford, UK formulated with GSK’s adjuvant AS01_B_ (Clinicaltrials.gov NCT02927145).

## Results

### Generation of a monoclonal *Drosophila* S2 stable cell line expressing the RH5.1 protein vaccine

**Fig. 1:**
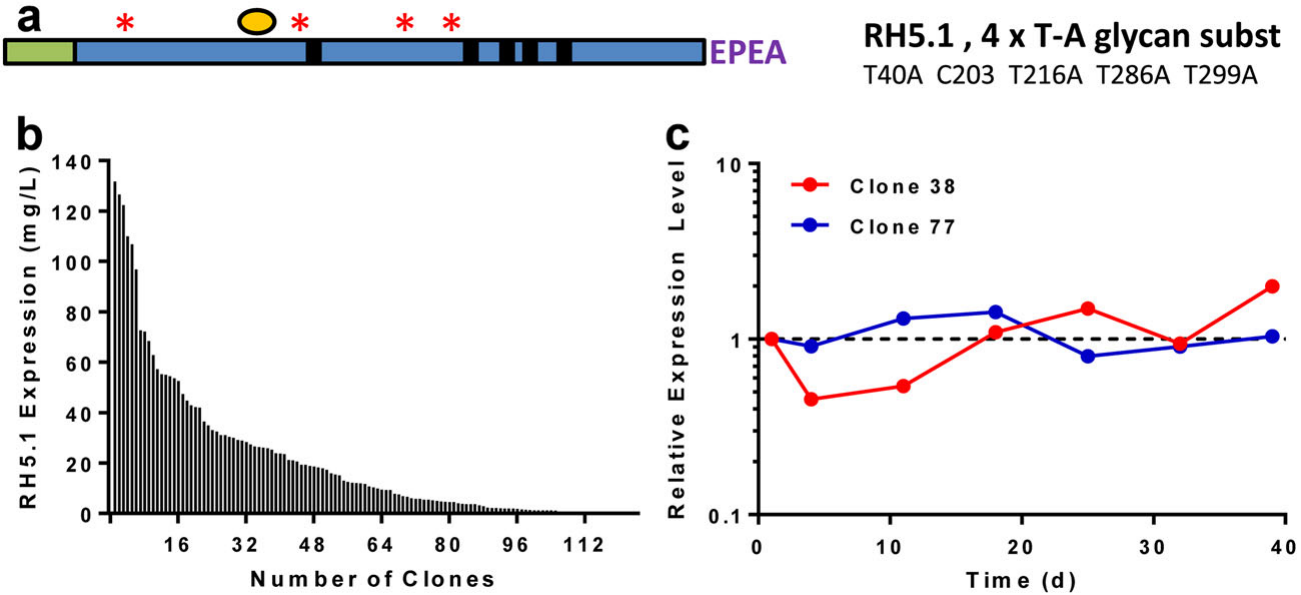
RH5.1 protein vaccine monoclonal stable S2 cell line generation. **a** Schematic of RH5.1 encoding from the N-terminus: a BiP insect signal peptide (green) followed by PfRH5 (aa E26-Q526) (blue), followed by a C-terminal four amino acid C-tag (EPEA). The protein was based on the *P. falciparum* 3D7 clone sequence, which has cysteine (C) at polymorphic position 203 (yellow circle). The other cysteine residues in PfRH5 are indicated by small black boxes (C224, C317, C329, C345, and C351). Threonine (T) to alanine (A) substitutions to remove N-linked glycan sequons are indicated by red asterisks. The predicted molecular weight (Mw) is 60.2 kDa. **b** A total of 124 single clones were selected and expanded in shake flasks. RH5.1 expression levels in the medium were assessed by quantitative ELISA. The distribution of expression levels is shown from highest through to lowest. **c** A total of 37 clonal cell lines were assessed for stability with respect to growth, viability, and productivity for up to 73 days. Example productivity data are shown for two clones (38 and 77) over a 39-day period. RH5.1 expression levels were assessed at indicated time points by ELISA, and are normalized to the concentration of RH5.1 measured on day 1 (set as 1.0)

We have previously reported the production of preclinical-grade full-length PfRH5 protein vaccines using polyclonal *Drosophila* S2 stable cell lines.^33,35^ For cGMP biomanufacture, we designed a final protein variant, termed “RH5.1” (Fig. 1a). The synthetic gene was subcloned into the pExpreS^2^−1 plasmid allowing for zeocin selection in transfected *Drosophila* S2 cells. Stable polyclonal cell lines were initially established and evaluated by ELISA to confirm RH5.1 expression. These cell lines were then cloned by limiting dilution to generate 124 RH5.1-producing clones. These were expanded to shake flasks and tested for RH5.1 protein yield by ELISA, where a large variation was observed (Fig. 1b). The 37 best expressing clones entered a stability evaluation scheme in order to determine their stability with respect to growth, viability (data not shown) and productivity (shown for two example clones in Fig. 1c). Expression levels were stable over time for both clones (maximum range 0.45–2.0 times the level measured on day 1). The highest producing stable clone was identified as clone 38, with productivity of ~100–150 mg/L by ELISA. Clone 38 cells were expanded and frozen in aliquots to produce the RCB.

The RCB for clone 38 was subsequently transferred to the CBF, University of Oxford, UK where one vial was thawed and used to produce 175 vials of a MCB, called “OxS2-RH5.1c38 MCB1.” Testing of the MCB confirmed its identity and sterility, and that it was negative for mycoplasma and spiroplasma. All other tests for adventitious agents met specified acceptance criteria (Table 1).

**Table 1:** Characterization and testing of the OxS2-RH5.1c38 MCB

Test	Specification	Result
Host-cell identity by random amplified polymorphic DNA (RAPD) assay	Positive	Positive
Sterility	Pass	Pass
Mycoplasma	Negative	Negative
Spiroplasma	Negative	Negative
Fluorescent product-enhanced reverse transcriptase (F-PERT) assay	Negative	Positive^a^
Electron microscopy examination of 200 median cell profiles	Negative	Positive^a^
Real-time PCR detection of porcine/bovine cirovirus (PCV)	Negative	Negative
Enhanced detection of a range of adventitious bovine and porcine viruses by nine CFR regulations using swine testis, bovine turbinate, and vero cells	Negative	Negative
Real-time PCR detection of flock house virus	Negative	Negative
Viral contamination in vitro cytotoxicity	Report result	Test item was not cytotoxic to any of the cell lines tested
Viral contamination in vitro: 28-day assay for detection of viral contaminants using four detector cell lines (MRC5, Vero, BHK, and C6/36)	Negative	Negative
Viral contamination in vivo: detection of toxicity of test article breakthrough (post neutralization) in suckling mice, adult mice, and guinea pigs	Report result	Test item free from toxic agents
Viral contamination in vivo: test for presence of inapparent viruses using suckling mice, adult mice, and guinea pigs	No viruses detected	No viruses detected
^a^HEK293 co-cultivation assay with F-PERT end point	Pass	Negative (pass)

Tests are listed with pre-defined specification and test results

^a^It was expected that the F-PERT assay for reverse transcriptase activity and TEM analysis of the MCB would be positive due to the presence of copia retrotransposons in *Drosophila* S2 cells. These cells have been observed to produce intracellular VLPs, but these have only ever been found to contain copia-derived protein and nucleic acid.^67^ For this reason, the HEK293 co-cultivation assay with F-PERT end point was initiated to test for retrovirus infectivity using mammalian cells as per guidelines in the European Pharmacopoeia

### Production and purification of the RH5.1 vaccine clinical batch

**Fig. 2:**
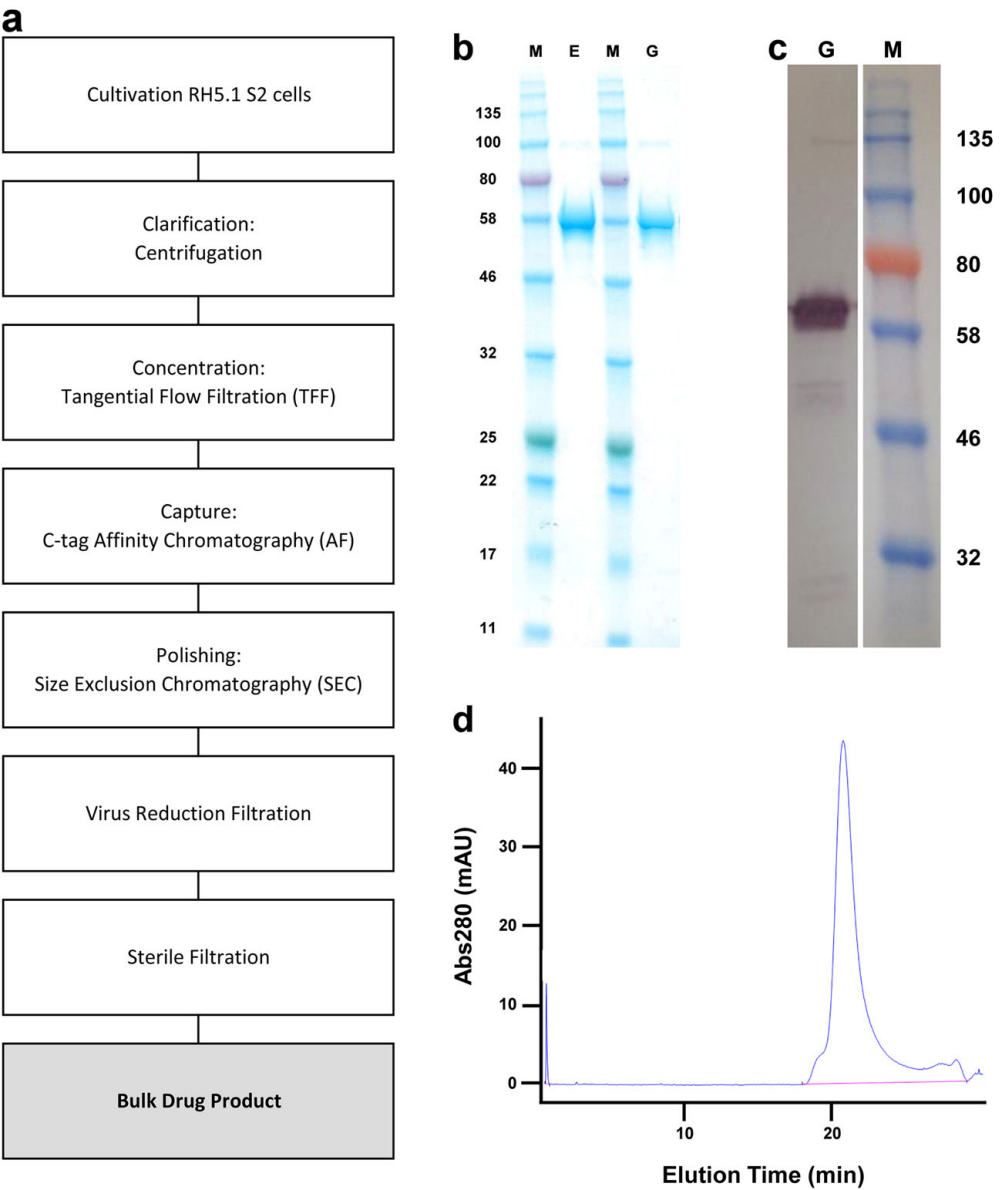
Analysis of the purified final RH5.1 drug product. **a** Overview of RH5.1 protein vaccine cGMP production process. **b** SDS-PAGE and **c** western blot (under reducing conditions) of the final RH5.1 drug product produced to cGMP (G) run alongside the comparator engineering batch (E). The western blot used the anti-PfRH5 4BA7 mouse mAb. Within each panel, the gels derive from the same experiment and were processed in parallel. **d** HPLC-SEC analysis of the final RH5.1 drug product to assess aggregation. M molecular weight markers

Extensive PD studies and production of an engineering batch of RH5.1 prior to cGMP manufacture had determined critical process parameters for each step in the final vaccine production process (Fig. 2a). The engineering batch was produced using the same RCB with identical process but at 1/10 the scale of the clinical batch. Each process step was scaled down according to manufacturers’ recommendations to ensure critical process parameters were consistent between the batches. In addition, a viral clearance study was undertaken in order to establish the ability of two keys steps in the cGMP biomanufacturing process, namely C-tag affinity chromatography and virus-reduction filtration, to effectively remove and/or inactivate (a) viruses, which are known to or could contaminate the starting materials, or (b) novel and unpredictable viruses. West Nile virus (WNV) and Porcine parvovirus (PPV) were used as models for these potential contaminants; WNV was selected as an example of an arbovirus (which can infect both insect and mammalian cells) and PPV as an example of a non-enveloped virus, which is highly resistant to chemical or physical destruction (Table 2). As indicated in the Committee for Proprietary Medicinal Products (CPMP) Note for Guidance on Virus Validation Studies (CPMP/BWP/268/95) from the European Medicines Agency, virus-reduction factors of ≥4 log_10_ are indicative of a clear effect for each particular virus. For steps where reduction factors of 1–3.9 log_10_ are reported, these steps contribute significantly to the overall removal and/or inactivation of virus and were therefore included in determination of the overall log_10_ reduction value. Overall, log_10_ reduction factors for the two steps investigated during this study were in excess of 7.5 log_10_, indicating that the cGMP process as a whole is effective in removing contaminating viruses.

**Table 2:** Viral clearance study

*C-tag affinity chromatography*	*Run 1*	*Run 2*
WNV	2.70 ± 0.43 log_10_	2.70 ± 0.41 log_10_
PPV	2.44 ± 0.56 log_10_	2.00 ± 0.38 log_10_
*Virus-reduction filtration*	*Run 1*	*Run 2*
WNV	≥5.00 ± 0.25 log_10_	≥5.17 ± 0.34 log_10_
PPV	≥5.77 ± 0.36 log_10_	≥5.69 ± 0.38 log_10_
*Overall reduction factors*		
WNV	≥7.70 ± 0.50 log_10_	
PPV	≥7.69 ± 0.54 log_10_	

Starting materials were spiked with the selected viruses and samples collected following the C-tag affinity chromatography or virus-reduction filtration process steps. Virus titer of each sample was determined by a 50% tissue culture infectious dose (TCID_50_) infectivity assay and the resultant virus log_10_ reduction factors are reported

cGMP production of the RH5.1 protein vaccine was thus performed at 25-L scale using ten 5-L shake flasks. Cell expansion occurred over 17 days with 7 cell passages. The cells showed ≥99% viability at the time of collection, and a density of 4.2 × 10^7^/mL. Subsequent downstream purification yielded 444 mg of RH5.1 protein (overall process yield of 17.7 mg/L). Approximately 94 mg of the final RH5.1 bulk drug product was filled into 500 vials as the clinical batch and stored at −80 °C. Test vials were subsequently thawed for analysis by SDS-PAGE. This confirmed a single main band at the expected size of 60.2 kDa, which appeared identical to the comparator engineering batch (Fig. 2b), with identity confirmed by western blot (Fig. 2c). Analysis by HPLC-SEC showed no detectable aggregation and purity >95% (Fig. 2d). The product therefore proceeded to further QC testing.

### QC testing of the RH5.1 vaccine clinical batch

Testing of the RH5.1 clinical batch confirmed its sterility, and that it was negative for mycoplasma and spiroplasma. All other tests for adventitious agents, endotoxin, protein concentration, appearance, pH, osmolality, residual host-cell DNA, and residual host-cell protein (tested by western blot) met specified acceptance criteria (Table 3). The product was also tested for residual anti-C-tag camelid single-chain antibody that could have leached from the affinity column—this test was passed with the product showing <2 ng/mL. In light of the results for the MCB testing (Table 1), the RH5.1 vaccine was also tested for copia retrotransposon gag protein by western blot, with results showing this to be negative.

**Table 3:** Characterization of the RH5.1 vaccine clinical batch

Test	Material	Specification	Result
Sterility test	Vialled product	Pass	Pass
Mycoplasma	Bulk harvest lot	Negative	Negative
Spiroplasma	Bulk harvest lot	Negative	Negative
Viral contamination in vitro: 28-day assay for detection of viral contaminants using three detector cell lines (MRC5, Vero, and C6/36)	Bulk harvest lot	Negative	Negative
Viral contamination in vivo: test for presence of inapparent viruses using suckling mice, adult mice, and guinea pigs	Bulk harvest lot	Negative	Negative
Abnormal toxicity test	Vialled product	Pass	Pass
Endotoxin	Vialled product	≤1400 EU/mL	0.482 EU/mL
Protein concentration	Vialled product	≥0.15 mg/mL ≤1.0 mg/mL	0.174 mg/mL
Appearance	Vialled product	Clear, colorless solution essentially free of visible particles	Pass
pH	Vialled product	Formulation buffer ± 1.0 pH unit	pH 7.14
Osmolality	Vialled product	200–600 mOsMol/kg	319 mOsMol/kg
Residual host-cell DNA	Bulk product	≤10 ng per dose	<180.0 pg/mL
Residual host-cell protein by western blot	Bulk product	Report result	Negative
Residual C-tag ligand	Bulk product	≤1 µg/mL^a^	<2 ng/mL
Copia gag western blot	Bulk product	For information only	Negative for copia protein at ~31 kDa
Identity by western blot	Vialled product	Positive for RH5.1	Positive for RH5.1
Purity by SDS-PAGE	Vialled product	RH5.1 bands >90% of total bands detected	>95%
HPLC-SEC	Vialled product	For information only	Pass

Tests are listed with pre-defined specification, the cGMP production material used for testing, and the test result. N-terminal protein sequencing was not done and was not required by the UK regulator (MHRA) for the RH5.1 vaccine to proceed to phase Ia clinical trial

^a^This specification was set to equate to <0.67% total protein

**Fig. 3:**
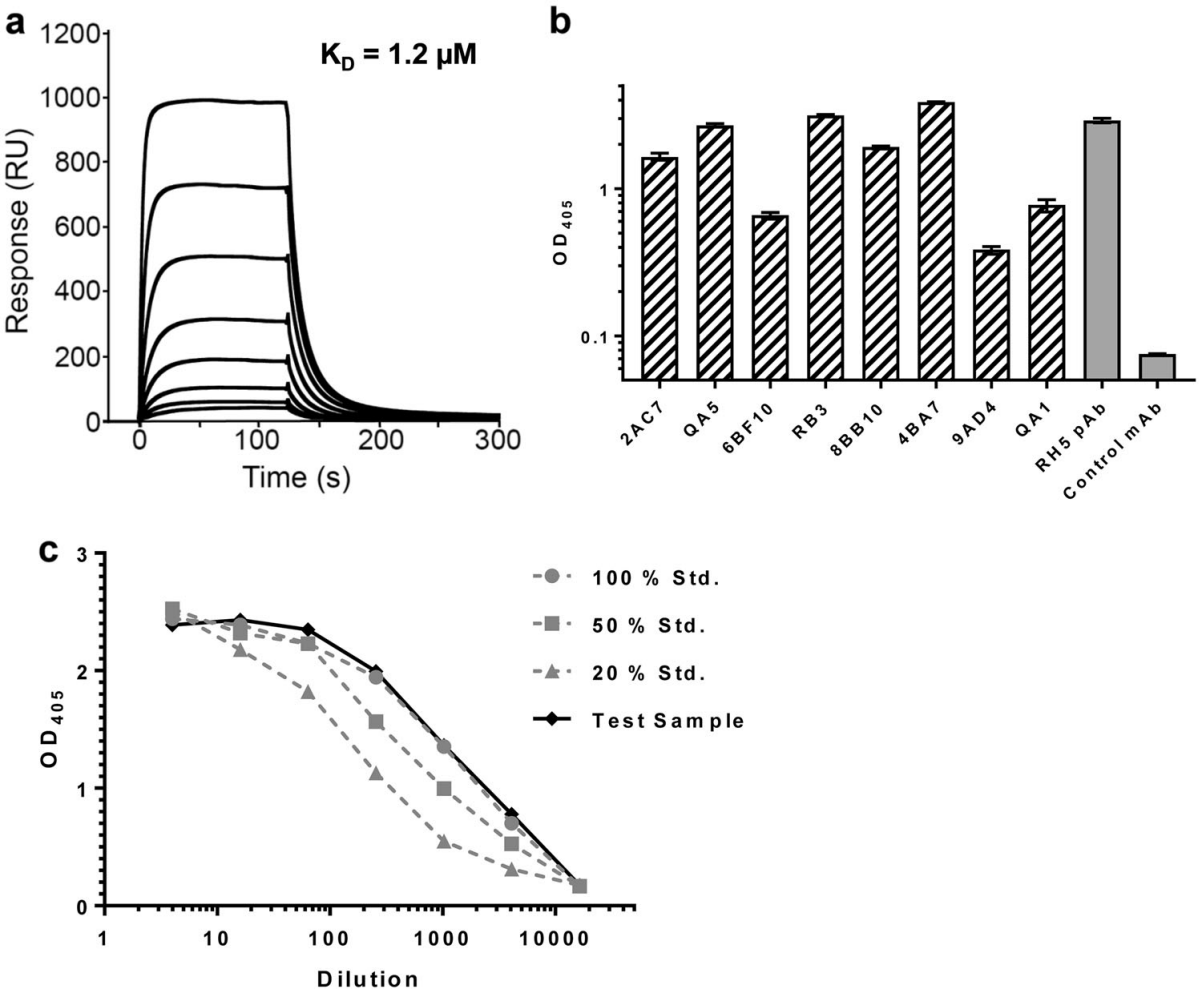
Characterization of RH5.1 clinical vaccine. **a** SPR analysis of the interaction of RH5.1 protein with basigin. **b** Anti-RH5.1 ELISA using a panel of eight PfRH5-specific mouse mAbs. Each sample was tested in triplicate. Bars show the mean plus range. **c** Potency ELISA using RH5.1 test sample versus RH5.1 protein standards (100, 50, and 20% concentration). Each point is the mean of triplicate readings. pAb mouse anti-PfRH5 polyclonal antibody serum control

The ability of RH5.1 protein to bind to recombinant basigin was subsequently analyzed by SPR, which measured an affinity of 1.2 µM, consistent with previously reported measurements of *K*_D_ = 1–2 µM^21,28,33,35^ (Fig. 3a). The ability of RH5.1 protein to be recognized by a panel of eight previously characterized mouse mAbs^37^ was also assessed by ELISA (Fig. 3b), confirming the presence of each epitope in the protein. Potency of the RH5.1 vaccine clinical batch was finally determined by a sandwich ELISA-based assay, using RH5.1 capture by the mouse mAb 4BA7 (which binds a linear peptide within the internal disordered loop of PfRH5), followed by detection with a non-competing, conformation-sensitive, neutralizing mAb 2AC7 (chimerized to have human IgG1 Fc in place of the parental mouse Fc). These data confirmed the clinical RH5.1 batch showed a relative potency that matched the 100% standard used in the assay (Fig. 3c).

### Stability of the RH5.1 vaccine clinical batch

**Fig. 4:**
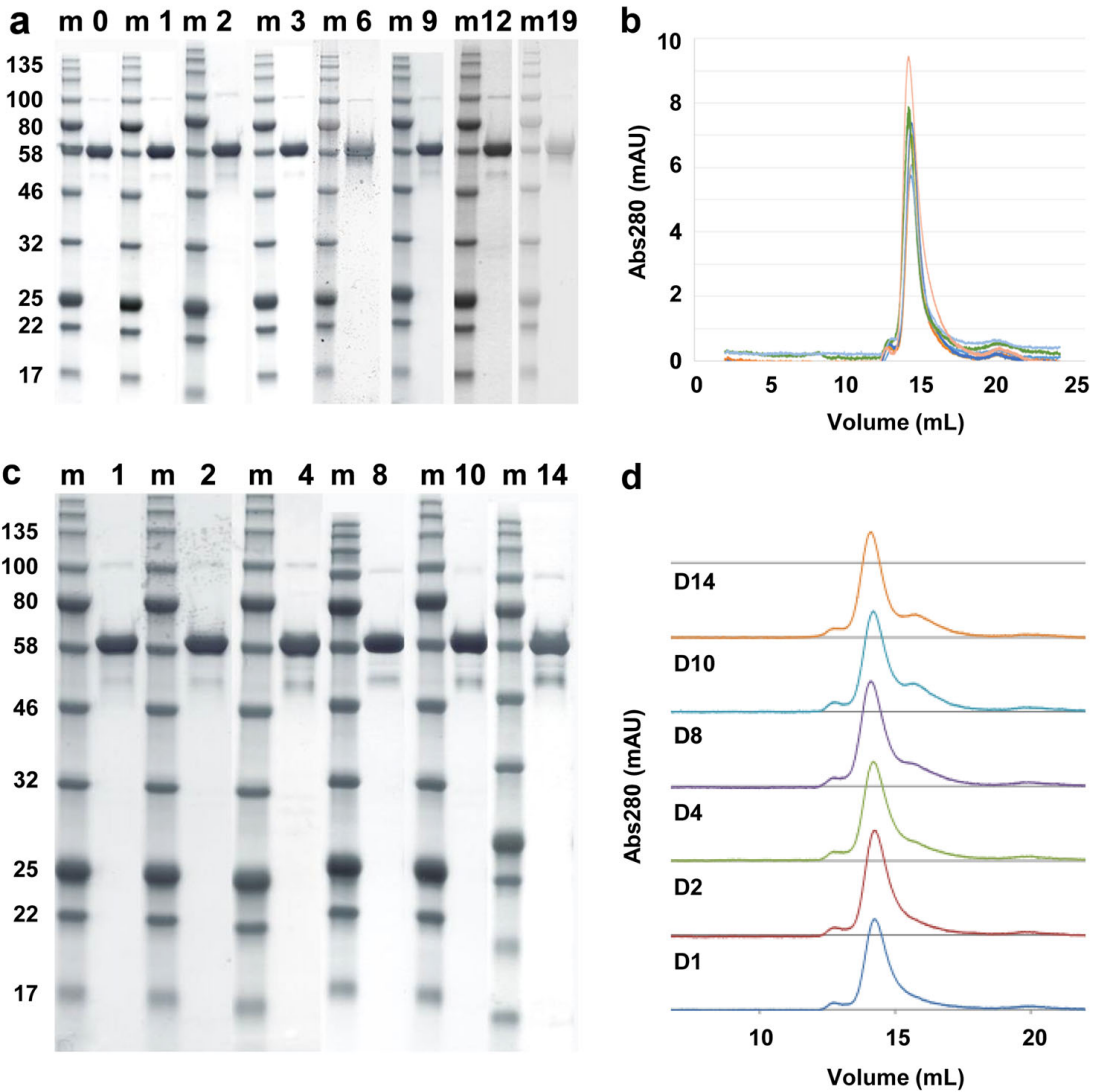
Stability testing of RH5.1 protein. RH5.1 protein vaccine was assessed for stability over time. The engineering batch was tested for **a** protein degradation by SDS-PAGE and **b** aggregation by analytical SEC following storage at −80 °C. Results are shown at the 0, 1, 2, 3, 6, 9, 12, and 19-month time points. In **b**, each colored line shows a different time point. The engineering batch was also tested for **c** protein degradation by SDS-PAGE and **d** aggregation by analytical SEC following storage at 4 °C as part of an accelerated stability study. Results are shown at the 1, 2, 4, 8, 10, and 14-day (D) time points. Within **a** and **c**, the gels for each time point derive from different experiments, but are shown aligned here for ease of comparison. m molecular weight markers

Stability studies were conducted on both the engineering batch and the final vialled product. The engineering batch was formulated in the same buffer as the cGMP-produced RH5.1 and stored at the planned storage temperature of −80 °C (range −70 to −85 °C), as well as at +4 °C (range 2–8 °C) to get accelerated insight of long-term product stability. The engineering batch was tested for protein degradation by SDS-PAGE (Fig. 4a), protein aggregation by analytical SEC (Fig. 4b), and identity and activity by a dot blot against the 2AC7 mAb (data not shown). All three test methods showed no detectable change in the RH5.1 protein over 19 months stored at −80 °C in comparison to the starting material (from the first assay time point). Similar results were obtained with the 14-day study conducted at 4 °C. SDS-PAGE analysis showed no additional lower molecular weight bands compared with the day 1 starting material, although a band similar in size to the natural ~45 kDa cleavage product of PfRH5^19,38^ became more pronounced by day 14 (Fig. 4c). Analytical SEC showed no protein aggregation, and a small shoulder by day 10–14 (in agreement with the appearance of some smaller product in the SDS-PAGE) (Fig. 4d). The potency sandwich ELISA (conducted on samples from days 8, 10, and 14) showed a relative potency that matched the 100% standard used in the assay (data not shown).

The clinical vaccine batch of RH5.1 is stored at −80 °C at the CBF, University of Oxford. Stability testing of the vialled product stored at −80 °C, as well as using an accelerated protocol at −20 °C, remains ongoing. At each time point, the product is tested for appearance (particles, color); pH; protein concentration; degradation by SDS-PAGE; identity by western blot; and potency by sandwich ELISA. At the time of writing, the product has been shown to be stable for at least 18 months, with no apparent changes in comparison to the starting material (from the first assay time point) (data not shown). Overall, these data suggest the cGMP-produced RH5.1 protein is stable and suitable for early-phase clinical testing.

### Characterization of the RH5.1/AS01_B_ vaccine formulation

**Fig. 5:**
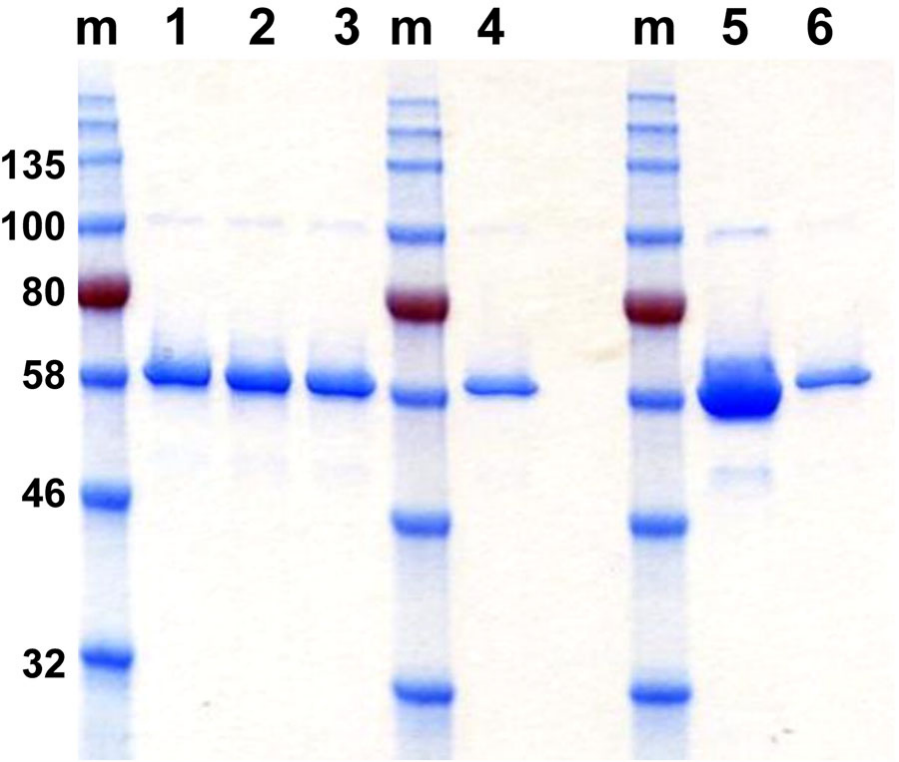
Characterization of the RH5.1/AS01_B_ vaccine formulation. Stability of the RH5.1 protein was assessed by SDS-PAGE at various time points after formulation in AS01_B_. Lanes 1–3 = immediate testing or after 1 and 4 h post mixing, respectively. Samples were also run on the same gel following testing of RH5.1 dilution using a mixing vial as required for clinical vaccine administration. Lane 4 = RH5.1 diluted 1:5 with 0.9% saline in a clinical mixing vial. Lane 5 = RH5.1 undiluted standard, and lane 6 = RH5.1 1:5 diluted standard. The gel in this figure derives from a single experiment with all samples processed in parallel

The vaccine product was subsequently assessed following formulation of RH5.1 protein (cGMP product) in the clinical adjuvant (final concentration 100 µg/mL RH5.1 in AS01_B_). The pH of the formulation was assessed immediately after mixing and was determined to be 6.39. Stability of the RH5.1 protein was also assessed by SDS-PAGE at various time points after formulation in AS01_B_. These data showed no significant change in the profile of RH5.1 immediately or after 1 and 4 h post mixing (Fig. 5, lanes 1–3). The first clinical trial of RH5.1/AS01_B_ also includes a 2 µg dose lead-in group, necessitating a 1:5 dilution of the RH5.1 cGMP product in the clinic using a mixing vial. This procedure was assessed here using the clinical SOP, in order to assess for potential loss of RH5.1 protein due to non-specific adsorption to the mixing vial. Analysis by SDS-PAGE showed no significant loss of RH5.1 following this procedure (Fig. 5, lanes 4–6).

Finally, we tested for changes in AS01_B_ particle size following addition of RH5.1. Analysis of the size distribution by scattered intensity for AS01_B_ alone, AS01_B_ + RH5.1 immediately post mixing, and AS01_B_ + RH5.1 stored for 1 h (to mimic bedside vaccine administration) showed that the size of liposomes was not changed post mixing, with median size of ~107 nm. As the concentration of RH5.1 is low compared to AS01_B_, no separate peak for RH5.1 was observed in the mixture. AS01_B_ was also monodisperse (with no detectable aggregation) shown by a mean polydispersity index <0.3 in all three conditions tested. Testing of the RH5.1 protein alone did not yield reliable data due to the low concentration of the vaccine product and small protein size (data not shown). Overall, these data confirmed that RH5.1 protein appeared stable in AS01_B_ adjuvant in the timeframe needed for clinical vaccine administration, and there was no discernible impact on the liposomal formulation of AS01_B_ following addition of RH5.1.

### Immunogenicity of RH5.1 formulated with AS01_B_

**Fig. 6:**
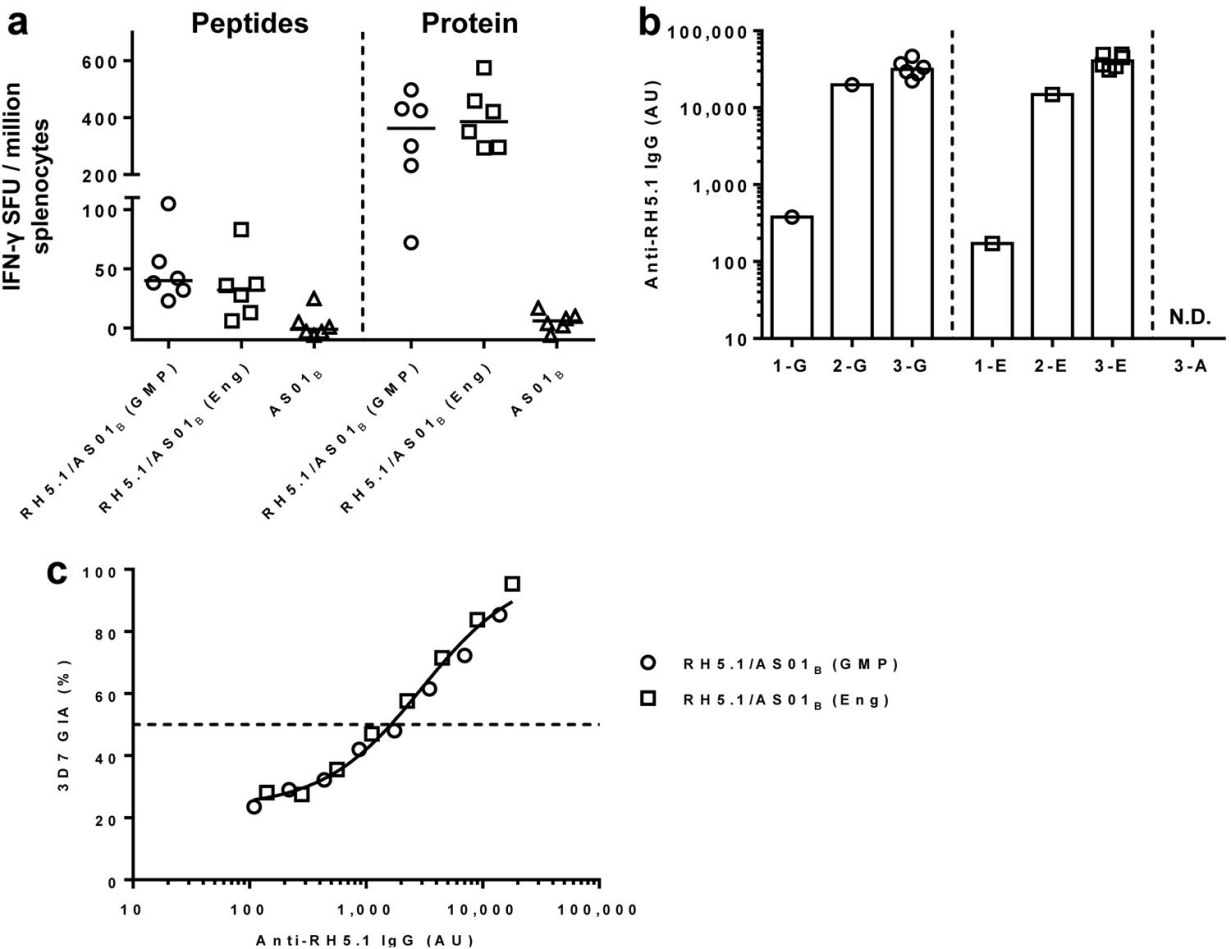
Immunological analysis of RH5.1/AS01_B_ in mice. **a** BALB/c mice (*n* = 6 per group) were immunized with 2 µg RH5.1/AS01_B_ using the cGMP-produced clinical vaccine batch (GMP) or the engineering batch of RH5.1 (Eng), or AS01_B_ alone. Two weeks after the last immunization, spleens were collected and T-cell responses were measured from spleen samples by ex vivo IFN-γ ELISpot following re-stimulation with RH5 peptides or RH5.1 protein. Median and individual data points are shown. **b** Serum IgG responses were measured by ELISA against RH5.1 using pooled serum samples taken 4 weeks after the first or second immunization using the cGMP-produced or engineering batches of RH5.1 (1-E, 1-G, 2-E, 2-G, respectively). Responses were measured in all mice 2 weeks after the third and final immunization (3-G, 3-E). There was no detectable IgG (N.D.) in any mouse following three immunizations with AS01_B_ alone (3-A). Individual and median responses are shown. **c** Functional GIA of purified IgG was assessed against 3D7 clone *P. falciparum* parasites. GIA is plotted against RH5.1 responses measured by ELISA in the purified IgG samples used for the assay, in order to assess quality of the vaccine-induced antibody response. The dashed line indicates 50% GIA. Non-linear least squares regression line is shown; *r*^2^ = 0.99, *n* = 16

Immunogenicity of RH5.1/AS01_B_ was assessed following i.m. immunization of BALB/c mice using protein from the RH5.1 engineering batch or the cGMP-produced RH5.1 clinical vaccine both formulated in AS01_B_. An extra adjuvant alone group was included as a negative control. Two weeks after the third and final immunization, spleens and sera were collected. An ex vivo IFN-γ ELISpot assay using splenocytes re-stimulated with recombinant RH5.1 protein or a pool of three peptides containing known H-2^d^ T-cell epitopes within PfRH5 showed similar cellular immunogenicity of both proteins with no significant difference as assessed by Mann–Whitney test (Fig. 6a). Similarly, the ELISA-detecting serum IgG responses against RH5.1, showed no significant difference between the two proteins as assessed by Mann–Whitney test. Finally, the IgG was purified from pooled sera and tested in a functional assay of GIA against 3D7 clone *P. falciparum* parasites. GIA was plotted against the RH5.1 responses measured by ELISA in the purified IgG used in the assay (Fig. 6c). There was no detectable GIA in the AS01_B_ only-immunized group (data not shown). These data showed that GIA was associated with anti-PfRH5 IgG as measured by ELISA, with a typical sigmoidal relationship, as observed in numerous studies with other antigens^39,40^ and both preclinical and clinical studies with PfRH5-based vaccines.^24,33,35^ The EC_50_s were also very similar for both the RH5.1 engineering batch and the clinical vaccine, confirming they elicit a very similar quality of antibody response. Overall, these data confirmed the immunogenicity of the RH5.1/AS01_B_ clinical vaccine and demonstrated that it could induce functional growth inhibitory antibodies.

## Discussion

Biomanufacture in accordance with cGMP is a critical step in the translation of any new vaccine into clinical testing. The field of malaria vaccines has historically seen many candidate antigens from all lifecycle stages produced for clinical trial as recombinant proteins in bacterial- and yeast-based expression platforms including *E. coli*,^41–47^ *Lactococcus lactis*,^48^ *Saccharomyces cerevisiae*^49,50^ and *Pichia pastoris*.^51–54^ However, generation of full-length PfRH5 protein proved particularly problematic in these heterologous expression platforms. Consequently, viral-vectored immunization led to the first promising results in animal models,^15^ whereby antigen is expressed in situ from virally infected muscle cells.^55^ Subsequently, a numer of groups reported production of preclinical-grade full-length PfRH5 protein using mammalian HEK293 cells,^21,56^ *E. coli*,^18,57^ baculovirus-infected insect cells^58,59^ and a wheatgerm cell-free expression platform,^60^ but not yeast-based systems. However, each approach faced different challenges for onward clinical development—these included the need for clinically incompatible C-terminal tags such as rat CD4 domains 3 and 4; production of insoluble protein within inclusion bodies; extremely low yield; or lack of a scalable cGMP-compliant process. Our subsequent demonstration that the ExpreS^2^ *D. melanogaster* S2 stable cell line system,^34^ coupled with C-tag affinity purification,^35^ was suitable for production of soluble full-length PfRH5 protein has now enabled the cGMP biomanufacture of a batch of RH5.1 clinical vaccine.

Prior to the cGMP biomanufacture campaign, a stable monoclonal RCB was generated by ExpreS^2^ion Biotechnologies under GLP conditions. Generation of a monoclonal cell line allowed for identification of a stable and high-producing clone, with RH5.1 levels >100 mg/L observed in 4% of the clones tested. This represented a 3–20-fold improvement over the expression levels observed from previously reported polyclonal S2 cell lines expressing PfRH5 protein variants,^33^ and allowed for viable progression to cGMP biomanufacture. Similar experiences in terms of isolating stable high-expressing monoclonal cell lines have been reported for a VAR2CSA-based *P. falciparum* vaccine for placental malaria produced in the same S2 cell system.^61^ Cell line expression level likely reflects a mixture of multiple transgene insertions following plasmid transfection as well as stochastic insertion loci within the S2 cell genome. Parameters that otherwise affect transgene expression from S2 cells remain poorly defined.

The monoclonal RCB was subsequently expanded under cGMP clean room conditions to produce the MCB. Extensive PD studies were undertaken to define the final upstream and downstream production process, alongside a viral clearance study to satisfy the regulatory requirement to demonstrate effective removal of potentially contaminating viruses. During these studies, we noted a consistent 100 kDa contaminant following C-tag column elution, which we identified by mass spectrometry as the *Drosophila* midline fasciclin protein.^62^ This protein does not have the C-terminal amino acids EPEA, suggesting a non-specific interaction with RH5.1 or the C-tag resin. This contaminant was different to a 38 kDa contaminant commonly observed when we previously purified PfRH5 proteins from polyclonal S2 cell lines,^33^ suggesting major contaminants can differ between clonal cell lines. However, this host-cell contaminant was effectively removed by the SEC polishing step, and the overall process ultimately yielded >400 mg highly pure RH5.1 protein from a 25-L batch produced in shake flasks. Ninety-four milligrams were subsequently filled into vials to produce the clinical vaccine lot, which on undergoing QC testing met all specified acceptance criteria.

This cGMP biomanufacture campaign also necessitated use of the C-tag resin, which is now commercially available. Notably, multiple different single-chain antibody-based affinity resins are available, known collectively as CaptureSelect™ technology, and the first product purified in this manner (an adeno-associated virus gene therapy product called alipogene tiparvovec (Glybera®) for lipoprotein lipase deficiency) has been licensed in Europe.^63,64^ Notably, use of the C-tag minimizes extra sequence in this vaccine to just four amino acids (excluding the BiP signal peptide that is cleaved from the protein during secretion from the S2 cells). Other malaria vaccine candidates have included much larger tags (for example two His6 tags) plus extra extraneous sequences (such as linkers or cloning sites). In some cases, these have totaled up to 29 non-pathogen amino acids fused to the desired antigen.^41–43^ These vaccines have been approved by natioanl regulators, including those in Europe and the US FDA, for clinical trials ranging from phase Ia through to phase IIb efficacy studies in African children with no apparent safety concerns.^29,65,66^

Testing of the monoclonal MCB showed that it was positive for reverse transcriptase activity with visible intracellular VLPs by TEM—this was expected due to the presence of copia retrotransposons in *Drosophila* S2 cells, an observation first made in 1972.^67^ *D. melanogaster* copia is in the *Hemivirus* genus of the family *Pseudoviridae*, and is a retrotransposon rather than a virus. The *Pseudoviridae* family of retrotransposons are present in invertebrates and fungi (notably including the Ty elements present in yeast such as *Saccharomyces*). There is no evidence that copia VLPs are transmissible to mammalian cells, as confirmed by a subsequent HEK293 co-cultivation assay for retrovirus infectivity as per guidelines in the European Pharmacopoeia. We therefore regarded copia VLPs as a form of host-cell protein not associated with any particular risks to human health. Moreover, it was highly likely that minimal crossover would occur from nuclear-located copia VLPs into the bulk harvest of the S2 cell supernatant, and even then a significant fold-reduction in copia VLP burden would occur during the downstream purification process as demonstrated by the viral clearance study. The final clinical batch of RH5.1 vaccine was also specifically tested for copia retrotransposon gag protein by western blot with results showing this to be negative, alongside testing for residual host-cell protein that met specified acceptance criteria.

Analysis of the RH5.1 clinical vaccine showed that it binds basigin with the expected affinity of 1–2 µM^21,28,33,35^ and that it is recognized by a panel of eight previously characterized mouse mAbs, many of which bind conformational epitopes.^37,68^ Stability testing of the RH5.1 clinical batch also confirmed the protein is stable at −80 °C and suitable for early-phase clinical testing. Notably, the ~45 kDa degradation product seen in the accelerated +4 °C stability study is comparable to the natural N-terminal cleavage product of PfRH5 found in parasite culture supernatants,^19,38^ and similar recombinant truncated proteins of PfRH5 also show strong immunogenicity and induction of growth inhibitory antibodies.^68,69^ We finally characterized RH5.1 following formulation with AS01_B_ adjuvant from GSK. These data showed that the protein was stable in the timeframe vaccine will be administered in the clinic, and that there was no discernible impact on the liposomal formulation of the adjuvant. Subsequent immunization of BALB/c mice confirmed that the cellular and humoral immunogenicity of the RH5.1 clinical batch was identical to that observed with engineering batch material produced in advance of the cGMP campaign. These vaccines elicited IgG that showed the same functional GIA against *P. falciparum* blood-stage parasites in vitro, in line with previous preclinical studies with PfRH5-based vaccines.^33,35^

The RH5.1/AS01_B_ vaccine has subsequently progressed through a preclinical toxicology study and was approved by the UK MHRA regulator for a phase I/IIa clinical trial in over 60 healthy adults in Oxford, UK, (Clinicaltrials.gov NCT02927145) using doses of 2, 10, and 50 µg. This trial will provide the first data on the safety, immunogenicity and efficacy of this vaccine formulation in humans (Minassian et al., in preparation). To date only a limited number of products produced to cGMP in *Drosophila* S2 cells have entered clinical testing—these include candidate vaccines for WNV and dengue virus^70,71^ as well as the VAR2CSA-based vaccine for malaria of pregnancy.^61^ Our data here further demonstrate the utility of the *Drosophila* S2 cell platform for cGMP-compliant biomanufacture and, alongside use of the C-tag purification technology, provide an alternative route for clinical translation of other “difficult-to-express” recombinant protein-based vaccines.

## Materials and methods

### Design and cloning of the RH5.1 protein vaccine

The design of the PfRH5 coding sequence within the RH5.1 protein vaccine has been described elsewhere, where it was reported as variant version 2.0.^33^ In brief, the protein encodes the full-length PfRH5 antigen (aa E26-Q526) based on the sequence of the 3D7 clone *P. falciparum* parasite, and all four putative N-linked glycosylation sequons (N-X-S/T) were mutated Thr to Ala—as performed for a previous PfRH5 protein vaccine produced in mammalian HEK293 cells and tested in rabbits^17,33^ and *Aotus* monkeys.^28^ The synthetic gene for RH5.1 was codon-optimized for expression in *D. melanogaster* and produced as a TSE-free product (GeneArt, Thermo Fisher Scientific). The gene also contained a Kozak sequence (GCC ACC) at the 5′ end, an N-terminal 18-aa Ig heavy chain binding protein (BiP) insect signal peptide (MKLCILLAVVAFVGLSLG) and a C-terminal four amino acid (EPEA) C-tag.^35^ This gene insert was subcloned by GeneArt into the pExpreS^2^−1 plasmid allowing for zeocin selection^33^ (ExpreS^2^ion Biotechnologies, Denmark) and verified by sequencing.

### Generation of the monoclonal *Drosophila* S2 stable cell line research cell bank

The *Drosophila* S2 cell line parental cell bank was established under Good Laboratory Practice (GLP) conditions by ExpreS^2^ion Biotechnologies, Denmark. A vial of the parental *Drosophila* S2 cell bank was resuscitated in EX-CELL 420 media (Sigma-Aldrich, UK) with 10% TSE-free certified fetal bovine serum (FBS) (provided by the CBF, University of Oxford) and expanded in shake flasks. Cells were transfected using ExpreS^2^ Insect-TRx5 reagent with the pExpreS^2^-1 plasmid encoding RH5.1 and cloned by limiting dilution. TSE-free certified FBS was present during the resuscitation, selection, and cloning process, but was removed by centrifugation during scale-up to shake flasks and replaced with serum-free medium, and no further FBS was used during the establishment or freezing of the Research Cell Bank (RCB). Stable polyclonal cell lines were selected by adding zeocin to the cells 24 h post transfection. The established polyclonal cell lines were evaluated by an anti-PfRH5 protein quantification ELISA (described in detail elsewhere^33^), and then diluted and seeded in 96-well plates and incubated. Clones from approved 96-well plates (plates containing less than one clone per three wells and visually confirmed to be single clones) were picked and transferred for further evaluation in 12-well plates and tissue culture flasks. A total of 124 RH5.1-producing clones were expanded to shake flasks and tested for protein expression by anti-PfRH5 protein quantification ELISA. The best expressing clones were further evaluated for their stability with respect to growth, viability, and productivity for up to 73 days (48 generations).

The highest producing stable clone was identified as clone 38. An ampoule of cells was frozen in CryoStor CS10 cryopreservation medium (Sigma-Aldrich, UK) shortly after establishment of clone 38 and resuscitated after the stability study. The cell line was resuscitated in serum-free and animal component-free EX-CELL 420 medium, and then expanded in ESF-AF serum-free and animal component-free medium (Expression Systems, USA) in shake flasks until a RCB of 30 vials containing 2.5 × 10^8^ cells per vial could be established. The cells were resuspended in Cryostor CS10 cryopreservation medium in 1 mL aliquots after centrifugation and stored at −80 °C, before transfer to −150 °C the following day.

### Generation and testing of the monoclonal *Drosophila* S2 stable cell line master cell bank

A single vial of the RCB clone 38 starting material was thawed for Master Cell Bank (MCB) generation in the cGMP clean room at the CBF, University of Oxford. The cells were expanded in shake flasks (25 °C, 130 r.p.m., and using ESF-AF serum-free and animal component-free medium) and maintained in the logarithmic phase of growth for 8 days until there were sufficient cells to lay down the MCB. Cells were frozen (1.5 × 10^8^ cells per vial) and stored at −80 °C and then transferred to liquid nitrogen vapor-phase storage. The MCB was named OxS2-RH5.1c38. All assays for testing of the MCB were performed by a contract research organization (CRO), SGS Vitrology, according to their standard operating procedures (SOP).

### cGMP production of recombinant RH5.1 final drug product

A vial of the MCB was expanded in culture to express the recombinant RH5.1 protein in the supernatant. The cells were scaled up from 20 mL to 25L (10 × 5-L shake flasks) over 17 days, while being maintained at 25 °C, 130 r.p.m. using ESF-AF serum-free and animal component-free medium with 2% production boost additive (PBA) (Expression Systems, USA) and 0.025% anti-foam (FoamAway, Thermo Fisher Scientific, UK) added at the last passage. The collected cell culture supernatant was then clarified by centrifugation (4000×*g*, 20 min, 20 °C), followed by 0.5/0.2 µm filtration (Opticap Express SHC, Merck Millipore, UK) for bioburden and aggregates reduction. Thereafter, the material was concentrated by a tangential flow filtration (TFF) system, fitted with Pellicon 3 Ultracel 10 kDa membrane (Merck Millipore, UK), in order to reduce the process volume for the subsequent steps. Design of single-use TFF assemblies was carried out with assistance from Merck Millipore, UK. Downstream process included a purification stack of a C-tag affinity chromatography^35^ and a polishing size exclusion chromatography (SEC), followed by a virus-reduction filtration, all performed on an ÄKTA Pilot system (GE Healthcare, UK). Suitable column sizes and operating conditions were determined during process development (PD). The ability to remove viral contaminants by the C-tag affinity chromatography and the virus-reduction filtration steps was also demonstrated in a viral clearance study, performed by a CRO (BioReliance) according to their SOPs. C-tag affinity resin (Thermo Fisher Scientific, UK) and SepFast GF-HS-L SEC resin (BioToolomics, UK) were packed into single-use columns, 50/30 (60 mL) and 50/1000 (2 L) respectively, by BioToolomics. Both columns were sanitized and bioburden tested after packing and before use. Concentrated culture supernatant was applied onto the C-tag affinity column. After washing, elution took place with 2 M MgCl_2_ and eluted fractions were pooled and stored at −80 °C. On the following day, C-tag column eluate was thawed and applied to the GF-HS-L SEC column, previously equilibrated with formulation buffer (20 mM Tris, 150 mM NaCl, pH 7.4 in water-for-injection). Fractions corresponding to the product peak, now in formulation buffer, were pooled, and 0.22 µm filtered for bioburden and aggregate reduction. The bulk purified lot was then filtered through a Viresolve Pro Modus 1.1 virus-reduction filter (Merck Millipore, UK), used with a pre-filter for optimal loading capacity, to generate the drug substance. The drug substance was then sterile filtered to generate the final bulk drug product. This was aseptically filled into glass vials to generate the final RH5.1 drug product, presented as a solution for injection. Each vial contained at least 165 µg in 0.95 mL formulation buffer.

### Recombinant RH5.1 clinical batch testing

The tests undertaken in Table 3 were performed by CROs (SGS Vitrology or RSSL Pharma) or by the CBF, University of Oxford according to their standard protocols and in accordance with the European Pharmacopeia. In brief, for sterility the test sample was aseptically transferred to soybean-casein digest medium and fluid thioglycollate medium. The broths were inspected for evidence of bacterial and fungal growth. The sterility assay was also qualified to ensure that the samples did not contain inhibitory factors. Mycoplasma was assayed using culture (indirect) and indicator cell culture (direct) methods, while spiroplasma was tested for using both agar and broth media.

Viral contamination was tested for in vitro by introduction of the test sample to different cell lines that allow for the detection of a wide range of human and animal viruses. Inoculated indicator cells (MRC5, Vero and C6/36) were observed for 28 days for morphological changes attributed to the growth of viral agents. The inoculated cells were passaged if required to assess for cytopathic effect (CPE). As some of the potential viral contaminants may not cause any morphological changes to the cells, the ability of inoculated cells to adsorb guinea pig erythrocytes to the cell surface (haemadsorption) was also assessed. For in vivo testing, adult mice, suckling mice, and guinea pigs were inoculated with test samples to look for extraneous agents. Embryonated eggs were not tested. For abnormal toxicity, the test sample was injected into five healthy mice and two healthy guinea pigs at the maximum proposed human dose. The animals were then monitored for ill health or death.

Bacterial endotoxin was assayed in test samples using the chromogenic kinetic method. Known amounts of endotoxin were tested in parallel with the test sample for an accurate determination of the level of bacterial endotoxin. The potential for interference by the test sample was examined by spiking in specified levels of endotoxin. Protein concentration was measured by absorbance at 280 nm (referenced at 320 nm) using a value for the extinction coefficient of 0.881 (0.1%). For appearance, the finished product was visually inspected using a liquid viewer with white and black backgrounds for the presence/absence of particles. The color of the vialled product was also assessed using color reference standards. pH was measured at 25.0 ± 1.0 °C after calibration of the pH meter with commercially available solutions in the appropriate pH range. Osmolality was measured after calibration of the osmometer using a 290 mOSm/kg standard. Each test sample was injected in triplicate and the mean value reported.

For residual host-cell DNA, the sample was extracted and then tested in a real-time PCR reaction containing target specific primers and a probe, alongside a range of positive controls of known host-cell DNA concentration. For residual host-cell protein analysis, the test sample and positive comparator sample (S2 cell supernatant) were separated by SDS-PAGE under reducing conditions followed by western blotting using an anti-S2 cell rat polyclonal antibody (ExpreS^2^ion Biotechnologies). Residual C-tag ligand was quantified using a commercially available CaptureSelect™ C-Tag Ligand Leakage ELISA Kit (Thermo Fisher Scientific). For analysis of the copia retrotransposon, a positive control sample of copia VLP was prepared by ExpreS^2^ion Biotechnologies from S2 cell nuclear material isolated by sucrose density centrifugation followed by hypotonic shock to release the VLP. This positive control sample and the test sample were then compared by SDS-PAGE under reducing conditions followed by western blotting using an anti-copia gag protein rabbit polyclonal antibody (raised against a synthetic peptide (CRILNNKNKENEKQVQTATSHG) from the C-terminus of the copia capsid protein). The antisera recognize a protein of ~31 kDa. RH5.1 identity was also confirmed by western blotting of the test sample following SDS-PAGE under reducing conditions and detection with the anti-PfRH5 4BA7 mouse monoclonal antibody (mAb).^37^ For purity analysis, test samples were separated in the same manner by SDS-PAGE and then visualized using a Coomassie visible stain. Bands were observed by densitometry and analyzed to determine purity.

### High-performance liquid chromatography-SEC

To assess for aggregation, test samples were separated isocratically using a Superdex 200 Increase 3.2/300 SEC column (GE Healthcare, UK) on an Agilent HPLC 1260 system using a flow rate of 0.075 mL/min. The molecular weight of any peak(s) detected was calculated by calibration against globular protein markers.

### Surface plasmon resonance

The production of recombinant basigin in Origami B (DE3) *E. coli* has been previously described.^68^ A section of the basigin gene encoding immunoglobin domains 1 and 2 of the short isoform (aa 22–205) was cloned with an N-terminal hexa-histidine (His6) tag followed by a tobacco etch virus (TEV) protease cleavage site. TEV cleavage leaves an additional glycine at the N-terminus from the cleavage site. Surface plasmon resonance (SPR) experiments were carried out using a BIAcore T200 instrument (GE Healthcare, UK). Experiments were performed at 20 °C in 10 mM HEPES (pH 7.4), 150 mM NaCl, 3 mM EDTA, 0.005% Tween-20, 2 mg/mL dextran, and 1 mg/mL salmon sperm DNA. Basigin was immobilized on a CM5 chip (GE Healthcare, UK) by amine coupling (GE Healthcare kit, UK) to a total of 950 Response Units (RU). A concentration series of RH5.1 protein (a two-fold dilution series from 2 µM) was injected over the basigin-coated chip for 120 s at 30 µL/min, followed by a 300 s dissociation time. The chip surface was then regenerated with 30 s of 2 M NaCl. Specific binding of RH5.1 protein was obtained by subtracting the response from a blank surface from that of the basigin-coated surface. The kinetic sensorgrams were fitted to a global 1:1 interaction model, allowing determination of the dissociation constant, *K*_D_, using BIAevaluation software 1.0 (GE Healthcare, UK).

### Potency and mAb ELISAs

RH5.1 protein was coated at 2 µg/mL with 50 µL per well onto a Maxisorp plate (Thermo Fisher Scientific, UK) and incubated at 4 °C overnight. The following day, the plates were washed six times with PBS/0.05% Tween-20 (PBS/T), before blocking with 200 µL per well 5% milk powder (Marvel) in PBS at room temperature (RT) for 1 h. After washing again six times in PBS/T, test mAbs were loaded (50 µL in triplicate) onto the plate at 5 µg/mL and incubated at RT for 2 h. The generation of eight PfRH5-specific mouse mAbs has been previously described.^37^ The positive control included mouse anti-PfRH5 polyclonal serum diluted 1:1000, and an irrelevant negative control mouse mAb. After a further 6 times wash, plates were incubated with goat anti-mouse IgG-alkaline phosphatase (Sigma-Aldrich, UK) diluted 1:1000 in 5% milk powder/PBS, using 50 µL per well at RT for 1 h. After a final six washes in PBS/T, plates were developed by addition of *p*-nitrophenyl phosphate substrate diluted in diethanolamine buffer (Thermo Fisher Scientific, UK). The optical density at 405 nm (OD_405_) was read using an Infinite F50 microplate reader (Tecan, Switzerland) and Magellan v7.0 software.

Potency of RH5.1 protein was assessed using a sandwich ELISA. Microplates were coated with the non-neutralizing murine mAb 4BA7 in PBS. After 6× washing in PBS/T, 200 µL per well Casein Blocker were added for 1 h at RT. Following another wash, RH5.1 protein standards (100, 50, and 20% concentration of the engineering batch) and test samples were added to the plate in a dilution series in triplicate for 1 h at RT. Following another wash, captured RH5.1 protein was detected for 1 h at RT using the chimeric human IgG1 mAb 2AC7 which recognizes a conformational epitope and is known to be highly neutralizing in the assay of GIA.^37^ Bound antibody was finally detected using anti-human IgG-alkaline phosphatase (Sigma-Aldrich, UK) and developed as above for the mAb ELISA. The relative potency of the test sample against the standards was then calculated using a four-parameter non-linear logistic regression model with calculated EC_50_ values. OD_405_ was read using an Infinite F50 microplate reader (Tecan, Switzerland) and Magellan v7.0 software.

### Stability testing of the engineering batch

For SDS-PAGE, protein test samples were heated to 95 °C for 10 min in Laemmli sample buffer containing 50 mM dithiothreitol (DTT). Electrophoresis was performed on a Criterion Any kD TGX gel (Bio-Rad Laboratories, UK) at 200 V for 45 min. The gels were then stained with Quick Coomassie Stain (Generon, UK) prior to imaging. For analytical SEC, test samples were separated isocratically on a Superdex 200 Increase 10/300 GL column (GE Healthcare, UK) using an ÄKTA Pure 25 system (GE Healthcare, UK) and in 20 mM Tris-HCl, 150 mM NaCl, pH 7.4 (TBS). For dot blots, RH5.1 protein was spotted in a dilution series onto nitrocellulose membrane (Bio-Rad Laboratories, UK) and air-dried. Afterwards, the membrane was processed in an iBind device (Thermo Fisher Scientific, UK), containing 2AC7 mouse mAb as primary at 1 µg/mL followed by an alkaline phosphatase-conjugated anti-mouse IgG secondary diluted 1:1000. Finally, the dot blot was developed with Sigmafast BCIP/NBT alkaline phosphatase substrate (Sigma-Aldrich, UK) prior to imaging.

### AS01_B_ formulation study

To characterize the RH5.1/AS01_B_ vaccine formulation, protein vaccine was thawed and added to vials of Adjuvant System AS01_B_ (provided by GSK) as per the clinical SOP. Samples were removed at defined time points (0, 1, and 4 h post mixing), heated to 95 °C for 10 min in reducing sample buffer and stored at −20 °C. SDS-PAGE was performed as for the stability testing.

### Dynamic light scattering

RH5.1 protein vaccine was mixed with AS01_B_ adjuvant to a final concentration of 63 µg/mL. DLS measurements were performed with at least 2 technical replicates of 11 measurements each running for >10 s for *Z*-average diameter. Malvern Instrument’s Zetasizer Nano and zeta cells (DTS1070) were used for the measurement, with parameters that were estimated to be closest to the actual formulation of AS01_B_ (dispersant PBS, refractive index 1.330, viscosity 0.8872cP). Briefly, the method detects and analyzes fluctuations in the intensity of light scattered by the particles when irradiated with red light (HeNe laser, wavelength *λ* 632.8 nm). Such fluctuations are detected at a backscattering angle of 173° and analyzed to obtain autocorrelation function using Malvern’s Zetasizer software version 7.11. The *Z*-average and polydispersity index values are provided from the cumulant analysis of the autocorrelation function by the software.

### Mice and immunizations

RH5.1 protein (either cGMP batch or engineering batch) was thawed for 15 min at RT before removal from vials using a syringe fitted with a 5 µm filter (Helapet IV1520 filter needle). This needle was then exchanged with a non-filtered 23 G needle and the protein transferred into another tube for dilution. Proteins were diluted in sterile PBS before mixing gently 1:1 with AS01_B_ adjuvant. The formulated vaccine was administered to mice within 1 h of mixing. All procedures on mice were performed in accordance with the terms of the UK Animals (Scientific Procedures) Act Project Licence and were approved by the University of Oxford Animal Welfare and Ethical Review Body. Female BALB/c (H-2^d^) mice aged 6–8 weeks were purchased from Harlan Laboratories (Oxfordshire, UK). Mice were anaesthetized with Isoflo (Abbot Animal Health, UK), and then immunized intramuscularly (i.m.) with 2 µg RH5.1 vaccine (50 µL in total) divided equally into each medial hamstring. Mice received three identical immunizations at 4-week intervals. Serum was harvested at stated time points from tail vein bleeds or by exsanguination under terminal anesthesia at the final harvest time point (2 weeks post-final boost).

### Ex vivo IFN-γ spleen enzyme-linked immunospot assay

Interferon-gamma (IFN-γ) ELISpot assays were performed using splenocytes as previously described.^10^ In brief, spleen cells were re-suspended at 1 × 10^7^ cells per mL in complete medium and plated at 50 μL cells per well. A 50 μL complete medium alone was added to control wells, and 50 µL re-stimulation in complete medium was added to duplicate test wells as follows: recombinant RH5.1 protein at a final concentration 5 µg/mL; or a pool of three peptides containing known H-2^d^ T-cell epitopes within PfRH5 (A7 = TYDKVKSKCNDIKNDLIATI [10 µg/mL]; C9 = NLNKKMGSYIYIDTIKFIHK [1 µg/mL]; and D9 = YIDTIKFIHKEMKHIFNRIE [1 µg/mL]) (NeoBiolab, USA). Results are expressed as spot forming units (SFU) per million splenocytes. Background responses in media-only wells were subtracted from those measured in re-stimulated wells.

### Anti-RH5.1 IgG enzyme-linked immunosorbent assay

Mouse anti-RH5.1 serum IgG responses were measured using a standardized ELISA according to previously described methodology^72,73^ and using a reference sample generated from high-titer sera pooled from PfRH5 vaccinated mice. A 1:8000 dilution of the reference sample gave an OD_405_ = 1.0, and thus this reference serum was taken to be 8000 arbitrary units (AU). Test samples were diluted appropriately so that their OD_405_ could be read off the linear part of the reference curve.

### Assay of growth inhibition activity against *P. falciparum*

Total IgG was purified from mouse sera using protein G columns (Pierce) and subsequently RBC depleted. The *P. falciparum* 3D7 clone laboratory-adapted line was maintained in continuous culture using fresh O+ erythrocytes at 2% hematocrit and synchronized by two incubations in 5% sorbitol 6–8 h apart. Synchronized trophozoites were adjusted to 0.4% parasitemia and then incubated for 44 h with the various IgG concentrations at 1% hematocrit. Final parasitemia was quantified by thin blood film of a tracker culture grown in parallel and growth inhibition was assessed by biochemical determination of parasite lactate dehydrogenase.^40^ Percentage growth inhibition is expressed relative to wells containing culture medium only (infection control, 0% growth inhibition) after subtraction of background signal (5 mM EDTA, uninfected control). The mean of the three replicate wells was taken to obtain the final data for each pooled mouse group at each tested IgG concentration. Experiments were performed twice with very similar results.

### Statistical analysis

Data were analyzed using GraphPad Prism version 6.07 for Windows (GraphPad Software Inc., California, USA). For the non-linear least squares regression, the equation: *Y* = bottom + (top−bottom)/(1 + 10((logEC_50_−X) × HillSlope)) was used with four-parameter curve and log_10_-transformed ELISA data, constrained at the top to <100% and at the bottom to >0% GIA. In the immunized mice, responses between the two test proteins were assessed by two-tailed Mann–Whitney test (as opposed to versus the negative control group) as the primary experimental question was to assess their comparability.

### Data and materials availability

Requests for data or materials should be addressed to the corresponding author.
